# Flow Cytometry Analysis Versus E-Cadherin Immunohistochemistry for the Diagnosis of Pure Erythroid Leukemia: A Case Report

**DOI:** 10.7759/cureus.9055

**Published:** 2020-07-07

**Authors:** Aziza AlSwayyed, Basmah Salamah, May Al-Moshary, Karrar Ahmad E Hussein Karrar, Adnan Khan

**Affiliations:** 1 Pathology and Clinical Laboratory Medicine, King Fahad Medical City, Riyadh, SAU; 2 Pathology and Clinical Laboratory Medicine, King Saud Hospital, Riyadh, SAU; 3 Pathology, Princess Nourah Bint Abdul Rahman University, Riyadh, SAU; 4 Oncology Center, King Fahad Medical City, Riyadh, SAU; 5 Pediatrics, Rehman Medical Institute, Peshawar, PAK

**Keywords:** aml-m6, pure erythroid leukemia, e-cadherin, glycophorin a

## Abstract

Pure erythroid leukemia (PEL) is a rare form of acute myeloid leukemia characterized by the neoplastic proliferation of erythroblasts. PEL is associated with inferior survival outcomes, particularly among patients harboring complex karyotype abnormalities. In this case, we present a 21-year-old Sudanese man who presented to our ER with a two-week history of fever, shortness of breath, fatigue, and exercise intolerance. He had no significant personal medical history or family history of malignancy. A bone marrow biopsy revealed hypercellularity and infiltration by cells with an immature appearance. A flow cytometry (FC) analysis of the bone marrow aspirate revealed that approximately 21% of the total nucleated cells were negative for CD45 and positive for CD71, glycophorin A, and CD36 but negative for myeloperoxidase (MPO), CD33, CD13, CD61, CD41, and other lymphoid and myeloid markers. Consistent with the microscopic analysis, <1% of the total cells were identified as CD34/CD13/CD117-positive myeloblasts. Notably, all stains (CD45, MPO, CD34, CD163, CD61, glycophorin A) were negative except E-cadherin, which positively stained >80% of the cells. Our findings suggested a differential diagnosis that included erythroid leukemia and myelodysplastic syndrome (MDS). The morphological, FC, immunohistochemistry, and cytogenetic findings strongly supported a diagnosis of PEL.

## Introduction

Pure erythroid leukemia (PEL) is a rare form of acute myeloid leukemia characterized by the neoplastic proliferation of erythroblasts [1]. Two types of erythroleukemia have been described by Di Guglielmo: Di Guglielmo syndrome, which involves myeloblasts and monoblasts, was described in 1917 while Di Guglielmo disease, which includes abnormal erythroblasts, was reported in 1928 [2-3]. Regarding diagnostic methods, flow cytometry (FC) is not particularly valuable for the the diagnosis of erythroleukemias because useful, specific markers for proerythroblasts have not yet been identified [4]. In recent years, however, epithelial calcium-dependent adhesion protein (E-cadherin) was found to be expressed on hematopoietic erythroid precursors in a developmentally regulated pattern, with a distinct expression on early erythroblasts followed by a gradual decrease during cellular maturation [5-6].

 Generally, PEL is associated with inferior survival outcomes, particularly among patients harboring complex karyotypic abnormalities. Therefore, allogeneic bone marrow transplant should be considered as the first line of treatment for suitable candidates once remission has been achieved, given the very high risk of relapse and mortality [7]. In this study, we review our experience with the challenging diagnosis of PEL in a patient with a low frequency of proerythroblasts, as well as the difficulty in meeting the current World Health Organization (WHO) diagnostic criteria for this erythroleukemia [8] and the importance of including E-cadherin as a potential diagnostic marker for PEL.

## Case presentation

A 21-year-old Sudanese man who resided in Yemen presented to our ER with a two-week history of fever, shortness of breath, fatigue, and exercise intolerance. He denied experiencing night sweats, weight loss, ecchymosis, or bleeding. He had no significant personal medical history or family history of malignancy, was not taking any medications, did not smoke, and did not abuse alcohol or drugs. He had previously been evaluated at a local hospital in Yemen, where he was found to have severe anemia and thrombocytopenia requiring repeated blood transfusions. Although his workup at that institution included bone marrow aspiration, no definite diagnosis was made. Upon physical examination, the patient was pale and febrile without any apparent distress. He did not exhibit petechiae, skin rash, or bleeding of gums. No lymphadenopathy or hepatosplenomegaly. The other physical examination findings were unremarkable.

 The initial hematological analysis revealed pancytopenia, with a white blood cell count of 2.32 x 109/L (3.90-11.00 x 109/L), absolute neutrophil count of 0.65 x 109/L (1.35-7.50 x 109/L), hemoglobin level of 9.70 g/dL (13.5-18 g/dL), red blood cell (RBC) count of 2.66 x 1012/L, mean corpuscular volume of 77.40 fl (75.0-95.0 fl), mean corpuscular hemoglobin level of 27.8 pg (24.0-30.0 pg), and platelet count of 3 x 109/L (155.0-435.0 x 109/L). The biochemical analysis revealed mildly elevated levels of lactate dehydrogenase and total bilirubin and renal function and liver function test results within normal limits. A peripheral smear showed slight anisopoikilocytosis with a few schistocytes and teardrop cells but no nucleated red blood cells or circulating blasts.

**Figure 1:**
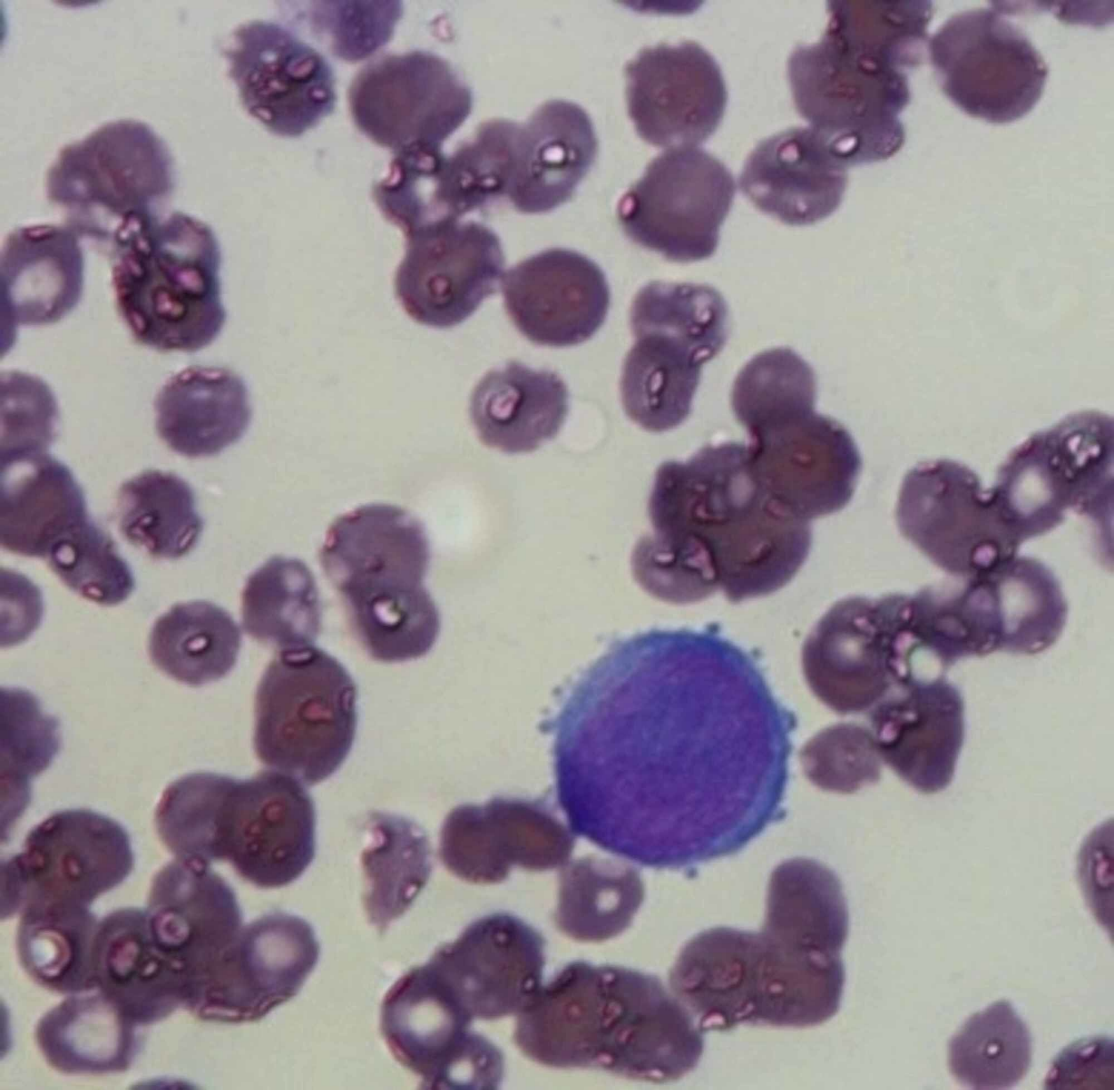
Proerythroblast with clear blebs.

 Examination of the initial bone marrow aspirate revealed diluted bone marrow comprising approximately 25% proerythroblasts and erythroid precursors (Figure 1).

**Figure 2:**
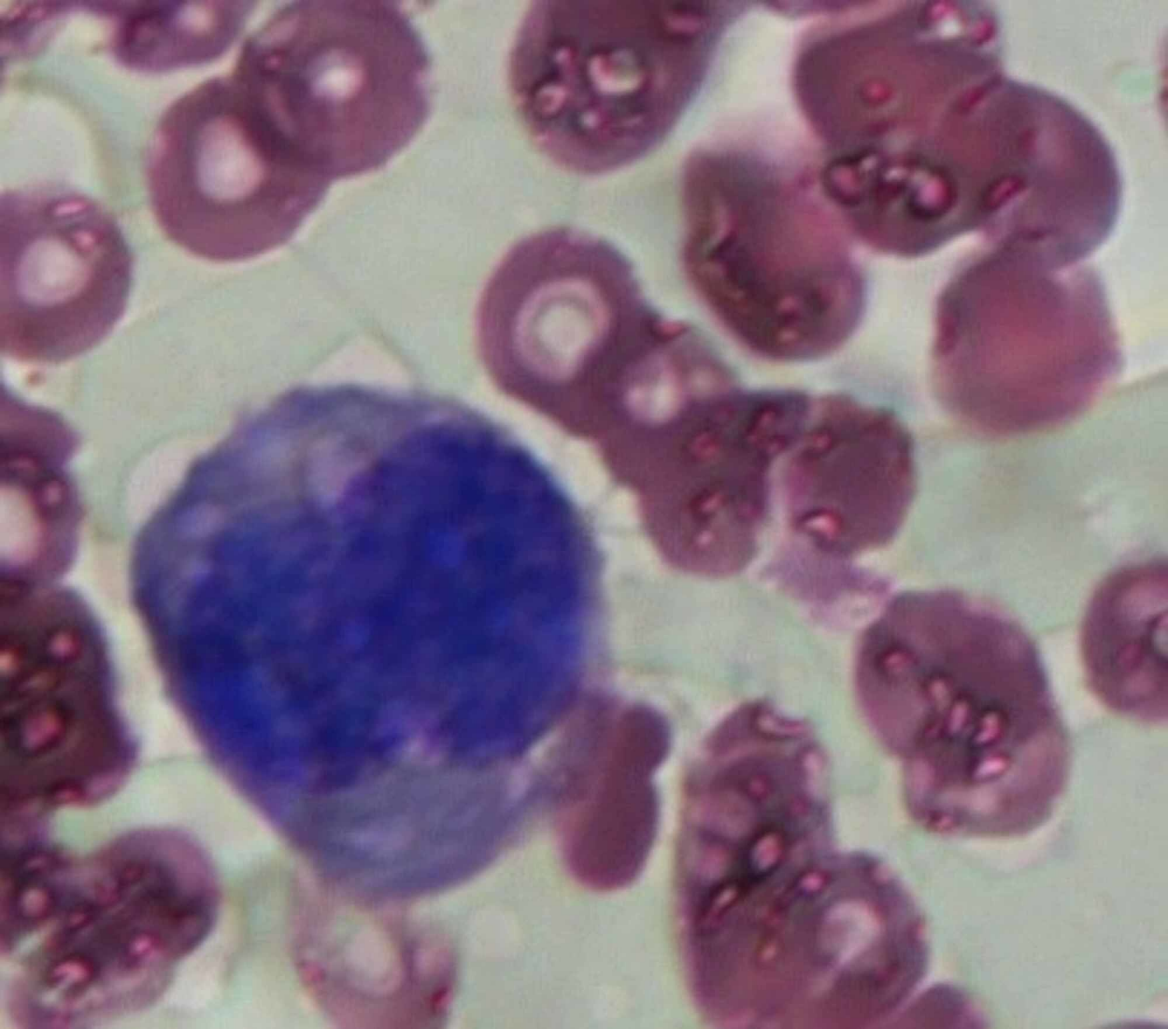
Large dysplastic erythroblast.

Erythroid precursors exhibiting a dysplastic morphology characterized by megaloblastic features and giant proerythroblasts with multinucleation were identified, in addition to lymphocytes (33%), myeloid cells (12%), monocytes (3%), plasma cells (1%), eosinophils, (1%), and myeloblasts (<1%) (Figure 2).

**Figure 3:**
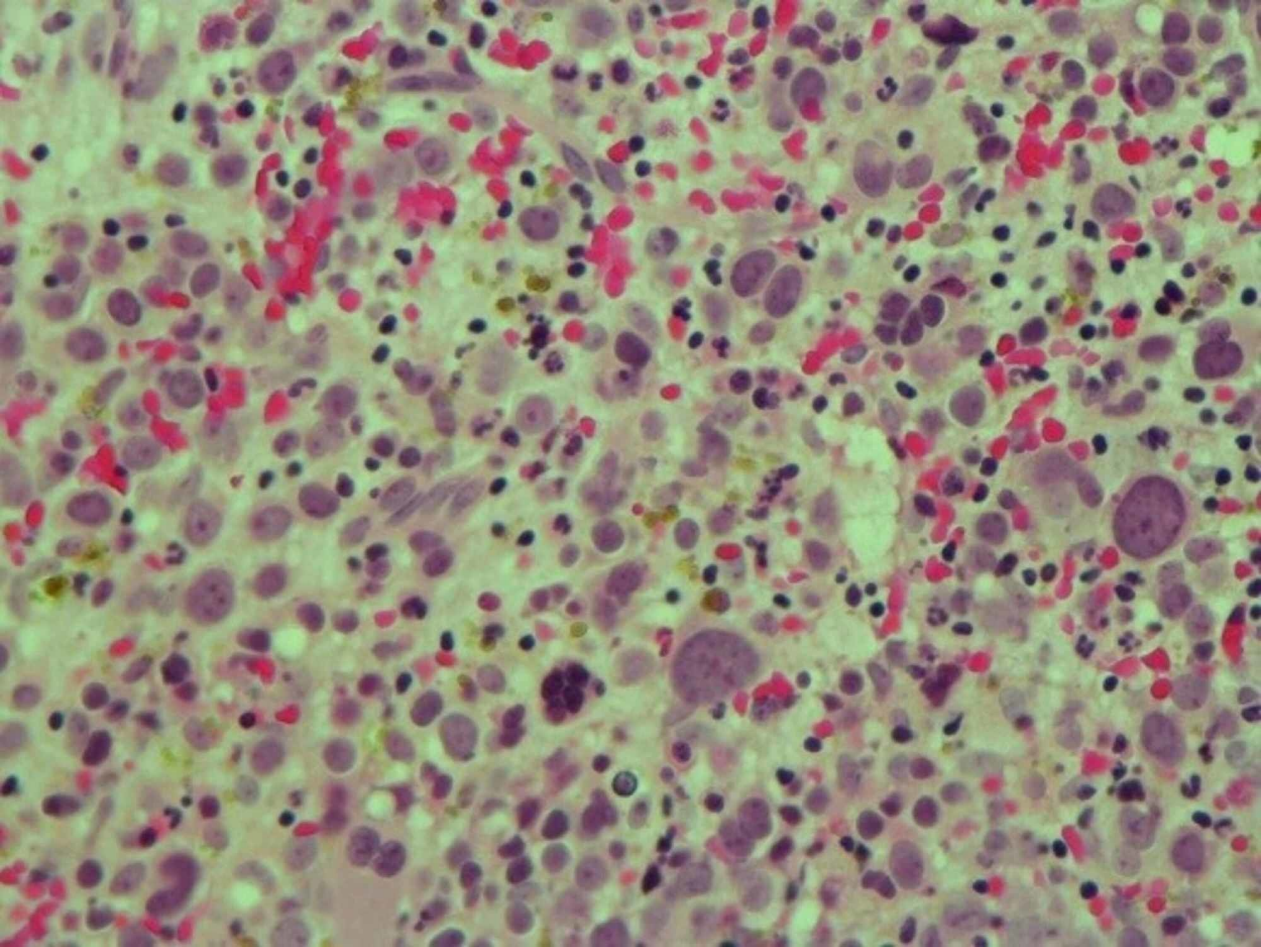
H&E stain showing interstitial proerythroblasts. H&E stain: hematoxylin and eosin stain

A FC analysis of the bone marrow aspirate revealed that approximately 21% of the total nucleated cells were negative for CD45 and positive for CD71 (30% of gated cells), glycophorin A (7% of gated cells), and CD36 (28% of gated cells) but negative for myeloperoxidase (MPO), CD33, CD13, CD61, CD41, and other lymphoid and myeloid markers. Consistent with the microscopic analysis, <1% of the total cells were identified as CD34/CD13/CD117-positive myeloblasts. A bone marrow biopsy revealed hypercellularity and infiltration by cells with an immature appearance (Figure 3).

**Figure 4:**
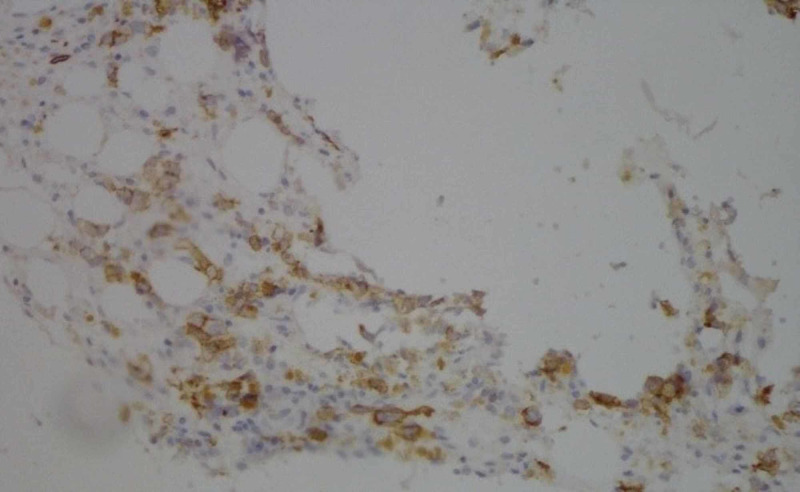
Immunohistochemistry for E-cadherin.

 Accordingly, an immunohistochemistry study was performed to characterize these immature cells. Notably, all stains (CD45, MPO, CD34, CD163, CD61, glycophorin A) were negative except E-cadherin, which positively stained >80% of the cells (Figure 4).

Regarding cytogenetic abnormalities, fluorescent in situ hybridization (FISH) analysis revealed the following complex clonal abnormalities in 63% of the scored nuclei: three extra copies of ABL1 (chromosome 9), PML (chromosome 15) L, RUNX1T1 (chromosome 8), MLL (chromosome 11), and five copies of RUNX1 (chromosome 21) in an average of 49% of scored cells. No evidence of deletion was observed on the long arms of chromosomes 5, 7, or 20, although these features are often seen in myelodysplastic syndrome. Molecular studies revealed a lack of mutations in CEBPA, FLT3 ITD, FLT3 TKD\T\, and NPM1, which are commonly seen in other types of acute myeloid leukemia.

 The patient was treated with an induction chemotherapy protocol comprising idarubicin (12 mg/m on days 1-3), cytarabine (100 mg/m continuous IV infusion on days 1-7), and etoposide (100 mg/m on days 1-5). The induction course was complicated by febrile neutropenia with negative cultures and grade II oral mucosal effects from which the patient recovered well. Subsequently, voriconazole treatment was escalated from a prophylactic to a therapeutic dose.

 On day 28, post-induction, bone marrow hypercellularity (i.e., cellularity = 30%) was observed. Approximately 15% of the proerythroblasts exhibited positive E-cadherin immunostaining, and aggregates of immature erythroid cells were observed. The patient was considered to have primary refractory disease and was administered a second-line chemotherapy regimen comprising 5-azacytidine (75 mg/m2 daily for five days), followed by fludarabine (80 mg/m2 daily on days 1-5), and cytarabine (2 g/m2 daily on days 1-5) with filgrastim for neutropenia. 

 Three weeks later, a bone marrow analysis again revealed hypercellularity (90%-100% cellularity) with regeneration of tri-lineage hematopoietic cells and 7% proerythroblasts. FC revealed an erythroid element frequency of 8%, with positivity for CD36 and CD71 and negativity for CD34, CD117, and glycophorin A. A FISH analysis revealed persistence of the complex clonal abnormality in an average of 42.9% of the scored nuclei as follows: one additional copy of BCR (9q43), PML (15q22), RUNX1T1 (8q22), and MLL (11q23) and three additional copies of RUNX1 (21q22).

 After an additional four weeks, the patient exhibited pancytopenia. A bone marrow analysis revealed approximately 80% cellularity, which comprised 53% E-cadherin-positive proerythroblasts. Given the high risk of relapse, the patient underwent an allogeneic cell transplant from a fully human leukocyte antigen-matched brother during the first remission but has unfortunately expired.

## Discussion

The WHO in 2016 [8] defined PEL as “a neoplastic proliferation of immature cells committed exclusively to the erythroid lineage (≥80% of bone marrow cells) with no evidence of a significant myeloblastic component.” However, in the study referred by 2008 WHO for the “description of PEL consisting of eight cases of minimally differentiated erythroleukemia, and the criteria for inclusion contain immature erythroblast ≥50% with <10% 'differentiating' forms” [9]. On the contrary, “Mazzella et al. [10]. have consistently used 30% pronormoblasts of erythroid precursors as the cutoff to define PEL.” In 2002, Domingo-Claros et al. studied 62 cases of acute erythroid neoplastic proliferation [11]. “Fifty-three cases were classified as AEL and two cases of PEL, leaving seven cases unclassifiable.” Two of the seven unclassifiable cases had undifferentiated erythroblasts of 42% and 40%, respectively, while our results investigated that the proerythroblasts and erythroid precursors (immature erythroblasts) were approximately 25%. In PEL patients, the peripheral blood smears show nonspecific changes in most cases, circulating erythroblasts may be observed in about 25% of patients, anemia and thrombocytopenia are commonly observed, which is also supported by our case [12].

In bone marrow aspirate, erythroid precursors with left shift and increased proerythroblasts are the abundant cells (>80%), which are the most immature identified erythroid precursor. They are medium to massive round nucleated blasts with, fine nuclear chromatin, occasional prominent nucleoli, the cytoplasm is dark blue and is agranular with different cytoplasmic vacuolization [13].

Flow cytometry is the primary technique for phenotyping acute erythroid leukemia (AEL), PEL, and other related conditions. The data obtained by FC can be comprehensive enough to completely subtype instances of leukemias/MDS into their myriad classes [14]. The possibility of characterizing individual cells according to their surface antigenicity by FC offers the chance to precisely subclassify the cells and sort them into separate populations [15]. “Glycophorin A positivity has been reported to be consistent with erythroid precursors, CD71 & partial CD36 positivity, also seen in erythroid precursors [16]. In the bone marrow, its expression appears mainly in immature erythroblasts, beta-sialoglycoprotein, carbonic anhydrase1, which can also be used to identify minimally differentiated M6 [17].” Our results revealed that a FC analysis of the bone marrow aspirate showed that approximately 21% of the total nucleated cells were negative for CD45 and positive for CD71 (30% of gated cells), glycophorin A (7% of gated cells), and CD36 (28% of gated cells) but negative for MPO, CD33, CD13, CD61, CD41, and other lymphoid and myeloid markers. Consistent with the microscopic analysis, <1% of the total cells were identified as CD34/CD13/CD117-positive myeloblasts.

“E-cadherin (epithelial cadherin) is a polypeptide protein that belongs to function is to facilitate cell-cell adhesion and recognition. E-cadherin has morphogenesis of cells during migration and acts as a tumor suppressor gene” [18]. In some solid tumors, such as breast cancer, loss of E-cadherin function is often associated with more invasive disease [19]. In our case, approximately 15% of the proerythroblasts exhibited positive E-cadherin immunostaining, and aggregates of immature erythroid cells were observed. In our study (FISH) analysis revealed the following complex clonal abnormalities in 63% of the scored nuclei: three extra copies of ABL1 (chromosome 9), PML (chromosome 15) L, RUNX1T1 (chromosome 8), MLL (chromosome 11), and five copies of RUNX1 (chromosome 21) in an average of 49% of scored cells.

## Conclusions

Our findings suggested a differential diagnosis that included erythroid leukemia and MDS. The morphological, FC, immunohistochemistry, and cytogenetic findings strongly supported a diagnosis of PEL.

## References

[1] Liu W, Hasserjian RP, Hu Y (2011). Pure erythroid leukemia: a reassessment of the entity using the 2008 World Health Organization classification. Mod Pathol.

[2] Di Guglielmo G (1917). Un caso di eritroleucemia Megacariociti in circolo e loro funzione piastrinopoietico. Richerche di ematologia I.

[3] DiGuglielmo G (1928). Le eritremie. Haematologica.

[4] Armeanu S, Muller CA, Klein G (2000). Involvement of E-cadherin in the development of erythroid cells; subject heading. Hematology.

[5] Redondo-Muñoz J, García-Pardo A, Teixidó J (2019). Molecular players in hematologic tumor cell trafficking. Front Immunol.

[6] Ohgami RS, Chisholm KM, Ma L (2014). E-Cadherin is a specific marker for erythroid differentiation and has utility, in combination with CD117 and CD34, for enumerating myeloblasts in hematopoietic neoplasms. Am J Clin Pathol.

[7] Latif N, Salazar E, Khan R (2010). The pure erythroleukemia: a case report and literature review. Clin Adv Hematol Oncol.

[8] Arber DA, Orazi A, Hasserjian R (2016). The 2016 revision to the World Health Organization classification of myeloid neoplasms and acute leukemia. Blood.

[9] Garand R, Duchayne E, Blanchard D (1995). Minimally differentiated erythroleukaemia (AML M6 Variant'): a rare subset of AML distinct from AML M6. Br J Haematol.

[10] Mazzella FM, Alvares C, Kowal-Vern A (2000). The acute erythroleukemias. Clin Lab Med.

[11] Domingo-Claros A, Larriba I, Rozman M (2002). Acute erythroid neoplastic proliferations. A biological study based on 62 patients. Haematologica.

[12] Wang SA, Hasserjian RP (2012). Erythroid proliferations in myeloid neoplasms. Hum Pathol.

[13] Hasserjian RP, Zuo Z, Garcia C (2010). Acute erythroid leukemia: a reassessment using criteria refined in the 2008 WHO classification. Blood.

[14] Sharma A, Buxi G, Walia R (2011). Childhood acute erythroleukemia diagnosis by flow cytometry. Indian J Pathol Microbiol.

[15] Bode-Lesniewska B (2016). Flow cytometry and effusions in lymphoproliferative processes and other hematologic neoplasias. Acta Cytol.

[16] Aljabry M (2015). Complex karyotype with novel translocation in pure erythroid leukemia patient. Hematol Rep.

[17] Acs G, LiVolsi VA (2001). Loss of membrane expression of E-cadherin in leukemic erythroblasts. Arch Pathol Lab Med.

[18] Rajwar YC, Jain N, Bhatia G (2015). Expression and significance of cadherins and its subtypes in development and progression of oral cancers: a review. J Clin Diagn Res.

[19] Canel M, Serrels A, Frame MC (2013). E-cadherin-integrin crosstalk in cancer invasion and metastasis. J Cell Sci.

